# 

**Published:** 1862-12

**Authors:** 


CLOSE OF THE VOLUME.

The present number closes the 16th volume of the Register, and in reference to the matter we have little to say. During the past year we have endeavored to perform our journalistic labors with a view to the best interests of the profession. We have endeavored to do what we could for its advancement. A little more assistance from our professional brethren would have rendered the work easier, and perhaps more satisfactory.

We much desire to have the aid and co-operation of those in the profession who have the ability to assist us. In the future we shall endeavor to perform our duty as we conceive for the best interests of the profession, and shall hope to merit and receive the aid of our dental brothers, both with the pen and purse.

### PHYSICIAN'S HAND-BOOK OF PRACTICE.

This is a case and appointment book for physicians fcr 1863, edited by William Olmer, M. D., and published by a W. A. Townsend, No. 39 Walker street, New York. It contains a classification of diseases, medical weights and measures and abbreviations, a large list of remedial agents, a list of incompatibles, directions in reference to baths, remarks upon extemporaneous prescriptions, poisons, their symptoms and treatment.

The record part of the book is arranged, first, with a department for keeping accounts, then a record list, then a department for general memorandums.

As a whole this is as full and complete as anything we have ever seen for the physician's use. Every physician of course uses something of the kind, and we have seen nothing better than this work. T.

### STEAM ENGINE.

We propose in the next number of the Register to give a description and an engraving of a little rotary engine, in use in our laboratory, for running dental lathes; it is a gem. T.

### PERSONAL.

Dr. William Taft, oldest son of our "T," has opened an office in West Greenville, Pennsylvania. He is a "chip of the old block," and a big chip at that—bigger than the block. Will. will succeed. No young man ever had better opportunities, and few have improved their opportunities as well. Indeed, he has succeeded. W.

☞ We intend to give in the January number of the Register a description of the method of lining badly fitting metallic plates, with vulcanized rubber, so as to restore the adaptation and still retain the articulation. We only propose to do this, provided some one else does not do it before as well or better than we can, which we hope may be case. T.

### TO OUR SUBSCRIBERS.

Those who wish to continue their subscription, for the next volume of the Dental Register, will please indicate the same to us immediately, as we always desire to know something about the extent of our subscription list before beginning the issue of a new volume; so please let us know your intentions at once.

There are a few subscribers on our list who are in arrears for the present volume; to most of these we granted indulgence because they expressly requested it. We hope they will at once remit the amount, for we could hardly be expected to send another volume unless this is done. Several things make this necessary. The cost of paper and publication is far more than formerly, and every thing is working up to a close cash system. We shall hope to hear from most or all of our subscribers, before or by the 20th of this month (December,) or as early as possible. J. TAFT.

### DIRECTORY OF HOSPITALS.

THE attention of clergymen, editors, and others is respectfully requested to the following notice, which is of interest to all who have friends in the army, and which it is therefore desirable should be widely published:

### DIRECTORY OF THE HOSPITALS.

The Sanitary Commission have established an office of information in regard to patients in the Hospitals of the District of Columbia, and of Frederick City, Maryland. By a refernce to books, which are corrected daily, an answer can, under ordinary circumstances be given by return mail to the following questions:

1st. Is———[giving name and regiment] at present in the Hospitals of the District or of Frederick City?

2d. If so, what is his proper address?

3d. What is the name of the Surgeon or Chaplain of the Hostal?

4th. If not in the hospital at present, has he recently been in hospital?

5th. If so, did he die in hospital, and at what date?

6th. If recently discharged from hospital, was he discharged from service?

7th. If not, what were his orders on leaving?

The Commission is prepared also to furnish more specific information as to the condition of any patient in the District hospitals, within twenty-four hours after a request to do so, from an officer of any of its corresponding societies.

The office of the Directory will be open daily from 8 o'clock A. M. to 8 o'clock, P. M., and accessible in urgent cases at any hour of the night.

The number of patients in these hospitals is about 25,000. If found to be practicable, the duty here undertaken locally by the Commission will be extended to include all the general hospitals in the country.

FRED. LAW OLMSTED.

*General Secretary.*

ADAMS HOUSE, 244 F STREET,

*Washington, D. C., November* 19, 1862.

SESSION 1861-'62.

### FACULTY.

T. L. BUCKINGHAM, D. D. S.,
Prof. of Chemistry and Metallurgy.

J. H. McQUILLEN, D. D. S.,
Prof. of Anatomy and Physiology.

WILLIAM CALVERT, D. D. S.,
Prof. of Mechanical Dentistry.

C. N. PIERCE, D. D. S.,
Prof. of Dental Physiology and Operative Dentistry

J. L. SUESSEROTT, D. D. S.,
Prof. of the Principles of Dental Surgery and Therapeutics.

D. H. GOODWILLIE, D. D. S.,
Demonstrator of Operative Dentistry.

J. J. GRIFFITH, D. D. S.,
Demonstrator of Mechanical Dentistry.

The regular Course will commence on the first Monday of November, and continue until the first of March ensuing.

During October, the Laboratory will be open, and a Clinical Lecture delivered every Saturday by one of the Professors, at 3 o'clock P. M.

The most ample facilities are furnished for a thorough course of practical instruction.

Tickets for the Course, Demonstrators' Tickets included, $100; Matriculation Fee $5. Diploma Fee $30. For further information, address W. CALVERT, DEAN,
133 North Eleventh Street, PHILADELPHIA.

### ROBERTS' OS--ARTIFICIEL.

Being desirous to fully test the merits of this new material as a substitute for AMALGAM, and all other cheap materials for filling teeth, I have put up a quantity in strong boxes containing from 75 to 100 fillings each, with directions, which will be sent postage free, on receipt of one dollar, to any dentist ordering of Messrs. Jones, White & Co., of New York, Philadelphia, Boston, or Chicago; or of Mr. Jno. T. Toland of Cincinnati, Ohio; or of the undersigned, who is using it daily in his practice, and considers it superior to Amalgam, Tin Foil, or any other coarse filling, or badly made gold filling.

Believing it to be a valuable acquisition to Dentistry, and that every member of the profession will value it as such, after a fair trial, I warrant every box sold to give satisfaction, or the money will be refunded on return of the article bought.

***C. H. ROBERTS, M. D,***

POUGHKEEPSIE, N. Y.

Jan. 1 y.

### THE WATTS

### CRYSTALIZED GOLD FOIL MANUFACTURING COMPANY,

### NEW YORK CITY.

A. J. Watts begs to inform his friends, the profession, that he is permanently engaged with the above Company, and is now preparing CRYSTAL GOLD FOIL and Soft or Non-Adhesive Foil, superior to any heretofore made by him. He now has every facility necessary for manufacturing, and all orders will meet with prompt attention. Please address

**A. J. WATTS, Agent,**

**Box 1296, New York.**

**To be had at all the Dental Depots.**

N. B. Beware of spurious imitations, as parties are endeavoring to sel he profession a different article by using my name.

None genuine unless marked "THE WATTS CRYSTALIZED GOLD FOIL MANUFACURING COMPANY."

### THE
### OHIO COLLEGE OF DENTAL SURGERY

### SESSION OF 1861–'62.

The regular Course of Lectures commences on the first Monday in November, and closes on Tuesday succeeding the fourth Monday of February.

### FACULTY:

JAMES TAYLOR, M. D., D. D. S.,
Professor of the Institutes of Medical and Dental Science.

W. H. ATKINSON, M. D., D. D. S.,
Professor of Anatomy and Histology.

J. TAFT, D. D. S.,
Professor of Operative Dentistry.

C. B. CHAPMAN, M. D.,
Professor of Chemistry and Physiology.

J. RICHARDSON, D. D. S.,
Professor of Mechanical Dentistry and Metallurgy.

Candidates for graduation must have attended two full courses of lectures,—the last in this school. Certificates of four years reputable Dental practice, or of one full course in any Dental or regular Medical College, will be considered as equivalent to the first course.

Half course tickets are issued to accommodate practitioners who can not leave their business for an entire session at once.

### Terms of Admission.

| | |
|---|---|
| Tickets for each entire Course of Lectures, | $60 00 |
| Matriculating Ticket, (paid but once,) | 10 00 |
| Diploma Fee, | 30 00 |
| Demonstrator's Fee, each session, | 5 00 |

An efficient Demonstrator of Mechanical and Operative Dentistry, will be secured before the opening of the school.

***JAMES TAYLOR, Dean.***

### BALTIMORE COLLEGE OF DENTAL SURGERY.

**TWENTY-FIRST ANNUAL SESSION 1860-61.**

### FACULTY.

CHAPIN A. HARRIS, D. D. S., M. D.
*Principles of Dental Science and Practice of Dental Surgery.*

THOMAS E. BOND, M. D.

*Therapeutics and Materia Medica.*

PHILIP H. AUSTEN, D. D. S., M. D.
*Mechanical Dentistry.*

REGINALD N. WRIGHT, M. D.
*Chemistry and Metallurgy.*

A. SNOWDEN PIGGOT, M. D.
*Anatomy and Physiology.*

CHRISTOPHER JOHNSTON, M. D.
*Microscopical and Comparative Anatomy of the teeth.*

FERDINAND J. S. GORGAS, D. D. S.,
*Demonstrator of Mechanical Dentistry.*

SAMUEL T. CHURCH, M. D., D. D. S.
*Demonstrator of Operative Dentistry.*

The twenty-first Annual Session will commence on the 1st. day of October, and close on the 1st of March.

The regular course of LECTURES will commence on the 1st of November.

The entire day during October, and the afternoon of each day during the rest of the session, will be devoted to Practical Dentistry and Anatomical Dissections.

The Infirmary has a large and rapidly increasing supply of patients, affording to the student unsurpassed facilities for the acquirement of a PRACTICAL knowledge of every Operation in Surgical Medical and Mechanical Dentistry.

Professors' Tickets, $100: Demonstrators' Tickets, $20; Matriculation Fee, $5; Diploma Fee, $30. Dissection Ticket, (optional), $10.

For further information, address **P. H. AUSTEN**, DEAN OF THE FACULTY,

79 North Charles Street, Baltimore.

---

### CHARLES ABBEY & SONS

MANUFACTURERS OF

### DENTISTS' FINE GOLD AND TIN FOIL,

***Nos. 228 and 230 Pear St.,***

PHILADELPHIA.

The attention of Dentists is invited to our FINE GOLD FOIL, which is prepared under our constant personal supervision. Our Nos. are 4, 5, 6, 8 and 10.

We are also manufacturing an ADHESIVE FINE GOLD FOIL, Nos. 4, 5 and 6.

ALL our Gold Foil is manufactured from ABSOLUTELY PURE GOLD, prepared expressly for the purpose with great care, by ourselves.

### Dentist's Refined Tin Foil constantly on hand.

CHARLES ABBEY,

WM. R. ABBEY,

CHAS. O. ABBEY.

### CONTENTS.

### DENTAL REGISTER,

### OF THE WEST.

Issued Monthly, at $3 a year in advance,

DEVOTED TO THE INTERESTS OF THE DENTAL PROFESSION.

**Edited by J. TAFT and GEO. WATT.**

The Volume commences in January and closes in December.

The Register being issued monthly is always fresh, therefore indispensable to the practitioner who desires to keep posted up with the new inventions and improvements which are daily developed. Neither expense nor effort will be spared to give value and variety to its contents. Articles will be illustrated with well executed engravings, whenever they will add to the interest or assist in explanation.

Its extensive circulation and frequency of issue makes it the BEST DENTAL ADVERTISING MEDIUM in the United States.

☞ CONTRIBUTIONS SOLICITED.

Address Original Communications to **Geo. Watt, Xenia, O.**

Exchanges to **J. Taft**, or **Dental Register, Cincinnati, Ohio.**

Business Communications to

J. TAFT, Publisher,
56 West Fourth Street, Cincinnati, O.

JNO. T. TOLAND, Proprietor,
38 West Fourth Street, Cincinnati, O.

Communications must reach the Editor as early as the 15th, to insure insertion in the next issue.

